# Evidence supporting the use of peptides and peptidomimetics as potential SARS-CoV-2 (COVID-19) therapeutics

**DOI:** 10.4155/fmc-2020-0180

**Published:** 2020-07-16

**Authors:** Sonya VanPatten, Mingzhu He, Ahmad Altiti, Kai F Cheng, Mustafa H Ghanem, Yousef Al-Abed

**Affiliations:** ^1^Center for Molecular Innovation, Feinstein Institute of Bioelectronic Medicine Feinstein Institutes for Medical Research, Northwell Health 350 Community Drive, Manhasset, NY 11030, USA; ^2^Robert S. Boas Center for Genomics & Human Genetics, Feinstein Institute of Molecular Medicine Feinstein Institutes for Medical Research, Northwell Health 350 Community Drive, Manhasset, NY 11030, USA

**Keywords:** ACE2, angiotensin-converting enzyme-2, antagonists, COVID-19, peptide, peptidomimetics, SARS-CoV, SARS-CoV-2, spike protein, viral entry

## Abstract

During a disease outbreak/pandemic situation such as COVID-19, researchers are in a prime position to identify and develop peptide-based therapies, which could be more rapidly and cost-effectively advanced into a clinical setting. One drawback of natural peptide drugs, however, is their proteolytic instability; peptidomimetics can help to overcome this caveat. In this review, we summarize peptide and peptide-based therapeutics that target one main entry pathway of severe acute respiratory syndrome coronavirus 2 (SARS-CoV-2), which involves the host angiotensin-converting enzyme-2 (ACE2) receptor and viral spike (S) protein interaction. Furthermore, we discuss the advantages of peptidomimetics and other potential targets that have been studied using peptide-based therapeutics for COVID-19.

Multiple zoonotic events since the turn of the millennium have resulted in disease outbreaks in the human population, including influenza A, H1N1, H5N1, H7N9, Zika virus and Ebola virus. Outbreaks due to members of the coronavirus family have been especially prominent in terms of transmission and mortality, beginning with the emergence of SARS-CoV in 2002–2003 [1,2], followed by middle east respiratory syndrome coronavirus (MERS-CoV) in 2012 [3] and SARS-CoV-2 (COVID-19) in late 2019 [4]. Key to the pathogenicity and infectivity of the coronavirus family in humans is the spike (S) protein, a large trimeric crown-like complex forming the namesake ‘corona’ as observed on electron microscopy (Figure 1). Gain of function studies on circulating strains of coronavirus in bats have evidenced the deterministic role of the S protein in selecting host species and tissue tropism [5]. The outbreak-associated coronaviruses SARS-CoV, MERS-CoV and SARS-CoV-2 share the commonality of binding host peptidases as their entry receptors; alternatively, other coronaviruses can recognize post-translational modifications such as glycans or adhesion molecules. Whereas MERS-CoV utilizes mainly dipeptidyl peptidase-4 (DPP4) [6], SARS-CoV and SARS-CoV-2 can enter human cells using angiotensin-converting enzyme-2 (ACE2) (Figures 2 & 3) [7,8] as the primary receptor [9,10]. Aside from recognition and entry through the primary receptor, secondary mechanisms exist for S protein mediated internalization, including through C-type lectins recognizing high-mannose glycans on the S protein [11]. Viral entry is also proposed to be possible through an endocytosis mechanism, with viral membrane fusion occurring in endosomes (reviewed in [9]).

**Figure 1.**
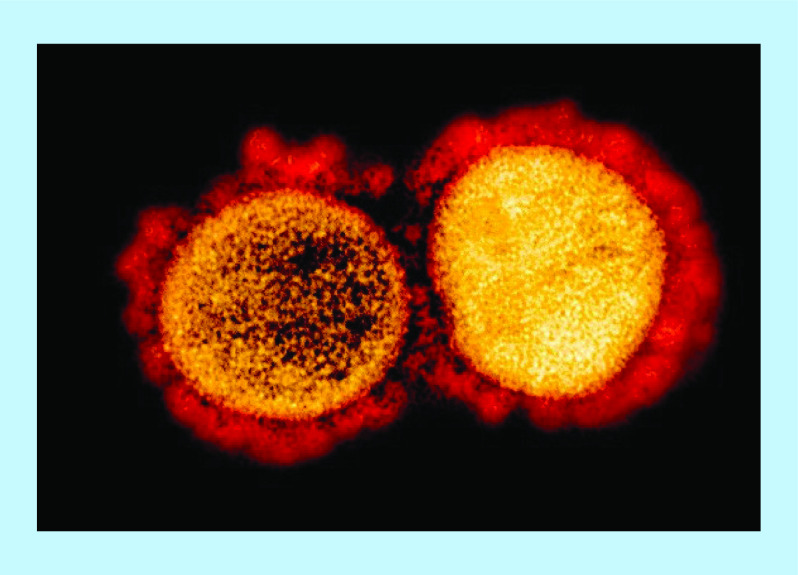
Transmission electron micrograph of SARS-CoV-2 virus particles, isolated from a patient. Image captured and color-enhanced at the NIAID IRF in Fort Detrick, Maryland. The spike protein (red) protrudes from the virions resulting in the namesake ‘corona’. Credit: NIAID-RML. IRF: Integrated research facility. Image used under creative commons license (https://www.flickr.com/photos/niaid/49645402917/in/album-72157712914621487).

**Figure 2.**
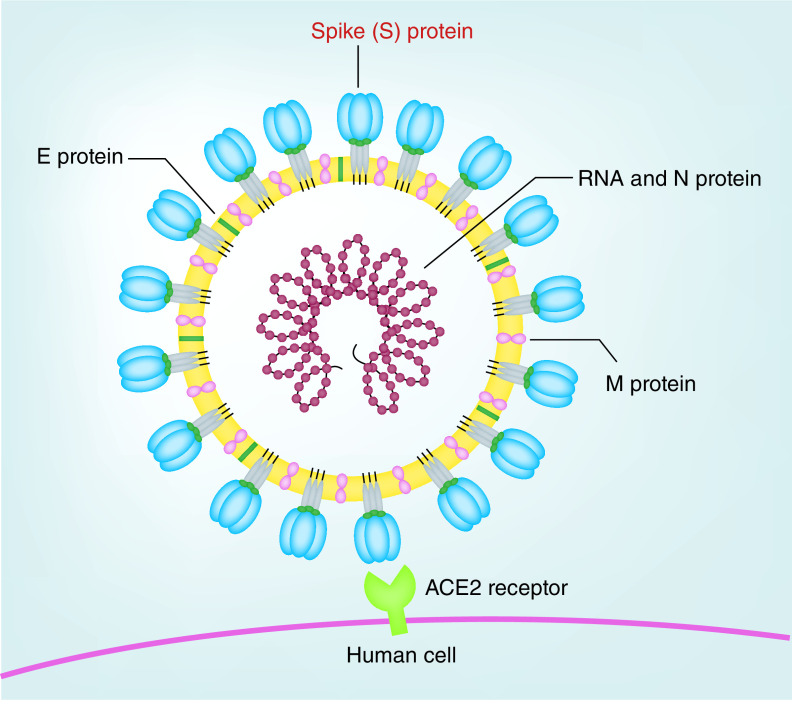
Representation of general structure of SARS-CoV's and host cell entry receptor ACE2. The major viral proteins and RNA strand are illustrated, these include the envelope **(E)** protein, nucleocapsid **(N)** protein, membrane **(M)** protein and spike **(S)** protein, along with the host entry receptor ACE2. The S protein-ACE2 interaction is a crucial mechanism of SARS-CoV entry into host cells.

**Figure 3.**
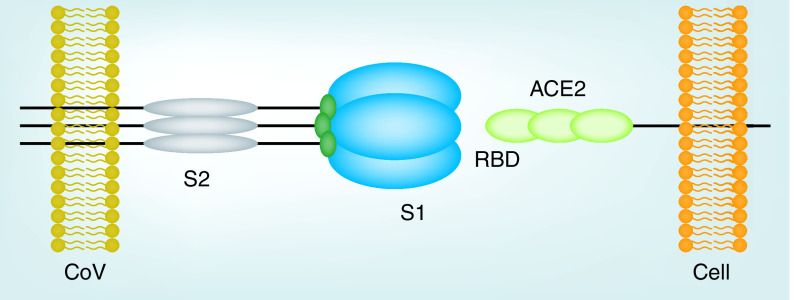
A close-up depiction of the S protein and host ACE2 receptor. The viral S protein (composed of S1 and S2 subunits) is a trimeric membrane-embedded glycoprotein. The RBD of the S1 region mediates initial binding with the host entry receptor ACE2, a transmembrane protein is represented in light green. The phospholipid bilayer of the viral particle is represented in yellow while host membrane phospholipid bilayer is represented in orange. RBD: Receptor-binding domain.

The S protein of the SARS-CoV and SARS-CoV-2 is a heavily glycosylated [12] ∼180–200 kDa Type I transmembrane protein with its N-terminus residing on the outside surface of the virus, and a short C-terminal region in the intramembrane viral space. The S protein comprises S1 and S2 subunits, which have a protease cleavage site between them (S1/S2), while another protease cleavage site exists in the S2 region (S2′). The S1 subunit mediates initial host receptor binding to ACE2, while the S2 region contains the membrane fusion machinery consisting of fusion peptide domains, and heptad-1 (HR1) and heptad-2 (HR2) domains that come together during host receptor binding to mediate the bridging of viral and host cell membranes (Figure 4) [13,14]. Amino acid (aa) residues within the C-terminal domain of the S1 subunit form the receptor-binding domain (RBD) responsible for receptor specificity (Figure 3) [15,16]. The RBD of the virus has been characterized in the sequence from the 2002–2003 outbreak of SARS-CoV and it resided in amino acids 318–510 of the S protein [17]. The minimal-binding domain was determined to be on a short loop on the receptor-binding domain consisting of 14 amino acid residues (424–494) of the S protein in these previous studies. The RBD sequence has been compared by the same group in SARS-CoV-2 and it resides in aa residues 331–524, furthermore, this report details sequence variation and conserved residues between SARS-CoV, MERS and SARS-CoV-2. The sequence similarity in the S2 domain is more conserved between SARS-CoV and SARS-CoV-2, making this region a better potential drug target for pan-SARS-CoV therapeutics [14,18].

**Figure 4.**
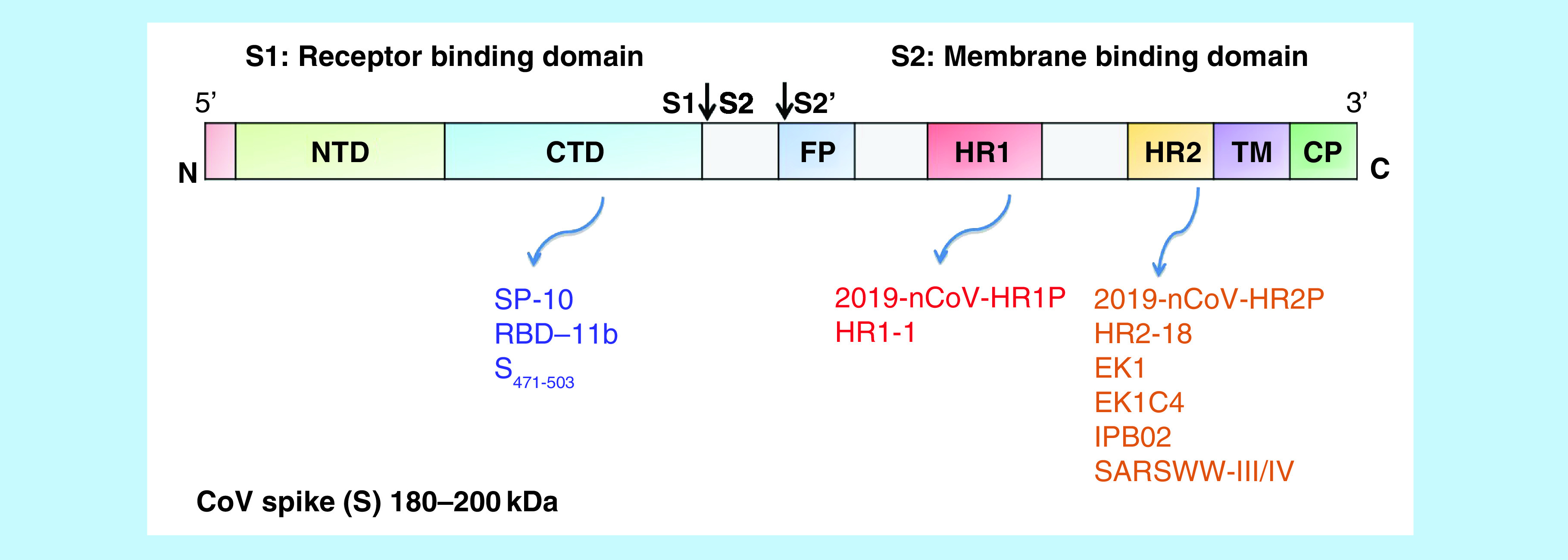
A 2D-representation of the major domains of the SARS-CoV S protein. The S protein is composed of the two subunits: S1 and S2. The S1 domain contains the NTD, the CTD, and is responsible for recognition and binding to the host cell receptor. The S2 domain, responsible for membrane fusion, contains the FP, the heptad repeat regions HR1 and HR2, the TM and CP tail. Two cleavage sites are indicated with arrows. The major peptide-based therapeutics, indicated below, are associated S protein regions. CP: Cytoplasmic; CTD: C-terminal domain; FP: Fusion peptide; NTD: N-terminal domain; TM: Transmembrane domain.

Studies have determined that after host receptor binding (ACE2), the viral S protein is processed by host cell proteases, including TMPRSS2 or likely other proteases [9,19]. Cleavage at the S1/S2 boundary releases the strain exerted on the S2 subunit by the S1 subunit, leading to a dramatic conformational change. The S2 subunit possesses fusion peptide (FP) domains, which help to mediate membrane binding between host and virus. The second protease cleavage site S2′ (Figure 3) exposes the FP permitting the formation of a six-helix bundle (composed of three alpha helixes each from the heptad repeat 1 and 2 regions [HR1 and HR2]), which mediates host/virus membrane fusion events, resulting in the delivery of the viral genetic material into the host cell. The tissue-specific distribution of proteases capable of processing the S protein further narrows the tropism of particular coronaviruses beyond the cell and organ-specific location of the primary receptor, ACE2 (reviewed in [19]).

The host receptor ACE2 is a type 1 transmembrane glycoprotein that functions enzymatically as a mono-carboxypeptidase. Its main physiologic function is to convert the biologically active vasoconstrictive and inflammatory peptide angiotensin-2 (8 aa) to angiotensin (1–7) by cleavage of the C-terminal amino acid. It can also act on angiotensin-1 (10 aa) (conversion to angiotensin (1–9), albeit with much less reported activity and affinity for this peptide substrate (400-fold less) [20]. Tissue expression occurs in numerous organs and cell types, but high expression was noted in lung alveolar epithelial cells and gut enterocytes [21], two important sites of SARS-CoV-2 exposure. The data on modulating endogenous ACE2 expression or its associated substrates are still in its infancy and no firm conclusions can be drawn until further studies are designed and carried out. This area is well reviewed by Perrotta *et al.* [22], and at present we feel the ACE2 substrates are less viable candidates for potential SARS therapeutics due to the potential for side effects, and they will not be addressed herein.

As of present, there are a few published studies looking at therapeutics for directly targeting the ACE2–S protein interaction that involve monoclonal antibodies, which have shown some promise in preclinical studies for SARS-CoV-2 [23,24], many others are beginning to appear in preprint servers. These potential therapeutics combined with the peptide and peptidomimetic candidates discussed herein, provide proof of concept for the potential value of this target in combating SARS-CoV-2.

## Advantages & potential of peptides & peptidomimetics as therapeutics

During an outbreak situation, traditional drug discovery is not an efficient option as this process is inherently slow to deal with the immense need for timely therapeutic solutions. There are several approaches that represent reasonable alternatives such as drug repurposing, vaccination and immunotherapy. Both immunotherapy and vaccination capitalize on peptide targets. Molecular details and the lessons learned from these strategies can be used to design and develop potential peptide-based therapeutics.

Peptides are smaller fragments of proteins and are preferable for ease of synthesis in terms of time and cost. In 2010, over 100 peptide drug candidates were reported in clinical trials and in 2019, three new peptide drugs were approved by the US FDA [25]. The advantages of peptides as drugs are rapid discovery, their specificity and affinity to desired targets, and low toxicity due to the limited possibility for accumulation in the body. Early on, peptides were considered poor drug candidates due to the inefficient and expensive synthesis processes, low bioavailability and limited stability against proteolysis by peptidases in the gastrointestinal tract and serum (t_1/2_ of natural and synthetic peptides are usually on the order of a few minutes). Thanks to technological advances, two chemical methodologies: solution-phase synthesis in 1953 [26] and solid-phase peptide synthesis in 1963 [27] dramatically dropped the cost of peptide manufacturing. The door of development for peptide-based therapeutics was widely opened by introducing peptides with varying sequence length, side-chain reactivity and degree of modification and incorporation of unnatural components.

To overcome the disadvantages of peptides as drug candidates, the field of peptidomimetics was introduced in early 1990s. A peptidomimetic candidate is usually based initially on a native peptide, which has been shown to inhibit protein interaction or function and which is then modified artificially to enhance bioavailability, improve transport through the blood–brain barrier (BBB), reduce the rate of clearance, and decrease degradation by peptidases [28,29]. There are numerous reported peptidomimetics [30], most of which were synthesized using altered solid-phase peptide synthesis methods. Some examples of peptidomimetics include, D-amino acid substitutions, those with reduced and functionalized amide bonds, peptoids, urea peptidomimetics, peptide sulfonamides, oligocarbamates, partial or full retro-inverso peptides, azapeptides, β-peptides and N-modified peptides.

In this review, we summarize the main peptide-based therapeutics that have been described for SARS-CoV-2 and SARS-CoV, which target the S protein–ACE2 interaction and entry process. Some of these are modified peptides (lipidated) or some form of peptidomimetic. Our aim was to focus mainly on the S protein–ACE2-mediated entry events (which includes subsequent membrane fusion steps), and we briefly highlight other potential targets for peptide/peptidomimetic-based inhibition, such as viral proteases.

## Investigations of peptide inhibitors targeting Spike–ACE2 interaction in COVID-19

Yan *et al.* [31] present cryo–electron microscopy structures of full-length human ACE2 with the RBD of the S protein of SARS-CoV-2, the RBD is recognized by the extracellular peptidase domain of ACE2 mainly through polar residues. These latest structural studies combined with previous research related to the RBD of SARS-CoV (2003) provide a basis for the development of therapeutics targeting this crucial interaction.

Another area of therapeutic interest is the targeting of the membrane fusion events. The S2 domain of the S protein, responsible triggering membrane fusion, includes the fusion peptide (FP), two heptad repeat regions (HR1 and HR2), a transmembrane (TM) domain and a short cytoplasmic (CP) tail (Figure 4). Following receptor binding, subsequent conformational changes in S2 creates a hydrophobic α-helical interface (six-helix bundle) as a result of binding between HR1 and HR2 regions [32]. The membrane fusion event is initiated when hydrophobic residues of the FP, insert in the host cell membrane. Due to the more conserved sequences between SARS-CoV and SARS-CoV-2 in the S2 domains, targeting SARS-CoV-2 entry (membrane fusion) could potentially have greater cross-functional success against coronavirus outbreaks.

Peptides derived from HR2 region were designed as SARS-CoV inhibitory peptides to disrupt fusion core formation. In studies examining SARS-CoV (2003) membrane fusion, exogenously-administered HR2 peptides blocked endogenous HR2 peptide binding with endogenous HR1 domains. The membrane fusion reaction was therefore inhibited, and pore formation was prevented. HR2-based antagonist peptides were also speculated to inhibit SARS-CoV-2, as suggested by the more highly conserved S2 subunits between SARS-CoV-2 and SARS-CoV, with 92.6 and 100% overall identity in HR1 and HR2 domains, respectively. In early 2020, Xia *et al.* reported their studies with the derivatives of SARS-CoV-2 HR2 peptides [33]. The group designed HR1- and HR2-derived peptides, termed 2019-nCoV-HR1P (aa924–965) and 2019-nCoV-HR2P (aa1168–1203), respectively (Table 1), and together with known pan-CoV fusion inhibitor (EK1 peptide) [34], explored their biological characteristics. Using a SARS-CoV-2 S-mediated cell–cell fusion assay, the researchers demonstrated that 2019-nCoV-HR2P and EK1 exhibited potent fusion-inhibitory activity with an IC_50_ of 0.18 and 0.19 μM, while 2019-nCoV-HR1P showed no significant inhibitory activity at concentrations up to 40 μM. Furthermore, both 2019-nCoV-HR2P and EK1 peptides showed significant inhibitory effect on SARS-CoV-2 pseudovirus infection in ACE2-expressing 293T cells, with IC_50_ values of 0.98 and 2.38 μM, respectively.

**Table 1.** Major peptide therapeutic candidates in development for SARS-CoV and SARS-CoV-2.

Potential peptide therapeutic	Target	Sequence/structure	Stage of development	Ref.
	**SARS-CoV-2 (COVID-19)**			
2019-nCoV-HR2P	HR1 domain of CoV S protein (MFM)	DISGINASVVNIQKEIDRLNEVAKNLNESLIDLQEL	Preclinical *in vitro* models	[33]
EK1	HR1 domain of CoV S protein (MFM)	SLDQINVTFLDLEYEMKLEEAIKLEESYIDLKEL	Preclinical *in vitro* models	[33]
EK1C4 (lipopeptide)	HR1 domain of CoV S protein (MFM)	(N)EK1-GSGSG-PEG4-(Chol)	Preclinical *in vitro* and *in vivo* models	[35]
IPB02	HR1 domain of CoV S protein (MFM)	ISGINASVVNIQKEIDRLNEVAKNLNESLIDLQELK(Chol)	Preclinical *in vitro* models	[36]
SBP1 (peptidase domain of ACE2)	S protein-RBD	IEEQAKTFLDKFNHEAEDLFYQS	Preclinical *in vitro* models	[37]
Inhibitor 1–4	S protein-RBD	ACE2-based peptides	Proposed in theory	[38]
	**SARS-CoV (COVID-2003)**			
EK1	HR2 domain of CoV S protein (MFM)	SLDQINVTFLDLEYEMKLEEAIKLEESYIDLKEL	Preclinical *in vitro* and *in vivo* models	[34]
HR1 and HR18	HR2 domain of S protein (MFM)	NGIGVTQNVLYENQKQIANQFNKAISQIQESLTTTSTA IQKEIDRLNEVAKNLNESLIDLQELGK	Preclinical *in vitro* models	[39,40]
P6	S protein-RBD	EEQAKTFLDKFNHEAEDLFYQSSGLGKGDFR	Preclinical *in vitro* models	[41]
SARS WW-III SARS WW-IV (derived from S2 subunit)	ACE2 receptor (MFM)	GYHLMSFPQAAP-HGVVFLHVTW GVFVFNGTSW-FITQRNFFS	Preclinical *in vitro* models	[42]
RBD-11b	ACE2 receptor	YKYRYL	Preclinical *in vitro* models	[43]
S_471–503_	ACE2 receptor	ALNCYWPLNDYGFYTTTGIGYQPYRWVLSFEL	Preclinical *in vitro* models	[44]
SP10	ACE2 receptor	STSQKSIVAYTM	Preclinical *in vitro* models	[45]

MFM: Membrane fusion mechanism.

Peptide-based inhibitors usually have a short half-life *in vivo*, which can critically limit their therapeutic effects. Emerging studies demonstrate that lipid-conjugated peptides exhibit dramatically increased antiviral potency and improved pharmacokinetics. To improve the inhibitory activity, Xia *et al.* applied conjugation chemistry to EK1, their work was published on 30th March in *Cell Research* [35]. Xia’s group generated a series of lipopeptides by linking cholesterol (Chol) through an amino acid linker (GSG) and/or polyethylene glycol (PEG) spacer to the C-terminus of EK1. Structure–activity relationship analysis suggested that ‘GSGSG-PEG4’ linker was optimal to bridge both parts of the conjugates and identified that EK1C4 exhibited the most potent inhibitory activity against SARS-CoV-2. EK1C4 was 241- and 149-fold more potent against SARS-CoV-2 S protein-mediated membrane fusion and pseudovirus infection with IC_50_‘s of 1.3 and 15.8 nM, respectively, as compared with EK1. EK1C4 has also been shown to be very effective against infection by SARS-CoV, MERS-CoV and other HCoVs. *In vivo*, intranasally applied EK1C4 showed strong protection of mice against HCoV-OC43 infection. Results of preinfection and postinfection treatment suggested that EK1C4 could be used to prevent and treat SARS-CoV-2 infection.

Recently, Zhu *et al.* [36] designed an HR2 sequence-based lipopeptide fusion inhibitor, named IPB02, which showed high activities in inhibiting SARS-CoV-2. Inhibition by IPB02 on the SARS-CoV-2 S protein-mediated cell–cell fusion was determined by a dual split protein-based cell fusion assay with an IC_50_ of 0.025 μM; inhibition on SARS-CoV-2 pseudovirus infection was confirmed by a single-cycle infection assay with IC_50_ values of 0.08 μM. By sequence truncation or extension, a group of lipopeptides around IPB02 was created. Structure activity relationship studies of IPB02 revealed the important roles of the N- and C-terminal amino acid motifs for their bindings and antiviral capacities. This group used circular dichroism spectroscopy to determine the structural properties of their cholesterol conjugated peptides. Circular dichroism results showed that the cholesterylated peptides exhibit significantly increased α-helical stability and target-binding affinity. This preprint report provides important information for understanding the entry pathway of SARS-CoV-2 and represents an ideal candidate for future optimization.

SARS-CoV-2 initiates its entry into human cells by binding to ACE2 via the RBD of its S protein. Thus, disrupting initial SARS-CoV-2 RBD binding to ACE2 is another strategy to inhibit the virus from entering human cells. Peptide-based entry inhibitors are attractive candidates to inhibit the RBD–ACE2 interaction and may serve as an effective antiviral drugs. Recently, Zhang *et al.* [37] demonstrated that the ACE2 peptidase domain (PD) α1 helix is important for binding SARS-CoV-2-RBD by using molecular dynamics simulations on the cocrystal structure of ACE2 and SARS-CoV-2-RBD [31]. The team chemically synthesized a natural 23-mer peptide (aa 21–43) from the human ACE2 (hACE2) α1 helix, termed SBP1. Kinetic binding assays using bio-layer interferometry was used to characterize the *in vitro* peptide–protein binding affinity. The results revealed that SBP1 strongly bound to SARS-CoV-2 RBD with a disassociation constant (K_D_) of 47 nM, which was comparable with that of the full-length hACE2 [46]. SBP1 is a first-in-class peptide binder to SARS-CoV-2-RBD, which may effectively disrupt the SARS-CoV-2–RBD/ACE2 interaction and block virus entry into human cells. However, the peptide needs to be tested in human cells and in animal models of SARS-CoV-2 infection.

Some researchers are using computer simulations to identify promising small molecules or peptides before conducting actual experiments in the lab. Applying computer-aided drug design techniques has emerged as an efficient means to quickly search potential therapeutics against SARS-CoV-2.

Han *et al.* [38] recently published a study in the journal *ACS Nano* that described how to use computer modeling to design compounds that mimic the spike protein’s natural target, ACE2. Using a recently solved X-ray crystal structure of the RBD of SARS-CoV-2 when it is bound to ACE2, researchers identified 15 amino acids from ACE2 that interact directly with the viral S protein. Then, four peptides (listed as inhibitors 1–4 in Table 1.) containing this domain, or parts thereof, were designed and encompassed two sequential α-helices extracted from the PD of ACE2. Molecular dynamic simulations revealed that the double α-helical peptides (such as inhibitors 2–4) were structurally stable and conformationally fitted to the RBD of SARS-CoV-2. Proposed in the report, at least one peptide inhibitor was able to specifically bind to the spike protein and prevent SARS-CoV-2–RBD/ACE2 interactions. Barh *et al.* [47] and Huang *et al.* [48] also shared their initial computational findings in preprints posted on *preprints* and *BioRxiv*, respectively. Using bioinformatics strategies, Barh *et al.* screened and designed several potential anti-S protein peptides that may limit viral attachment and entry. Binding to SARS-CoV-2 S protein RBD with the peptides was determined by the HPEPDOCK protein–peptide docking server. Ten peptides were predicted as attractive therapeutics against SARS-CoV-2. Huang’s group designed thousands of peptide binders that showed binding activity to SARS-CoV-2 in computational experiments. To enhance peptides’ binding affinity to SARS-CoV-2 RBD, researchers redesigned the peptide sequence by applying structural bioinformatics and sequence logo analyses. The top designed peptide binders were selected to run further experimental validations. Thus, computational approaches may lead us to find potential inhibitory peptides against SARS-CoV-2, but *in vitro*, and *in vivo* experiments are still required to evaluate and ensure their potential therapeutic efficacy.

## Peptide-based antagonists from SARS-CoV (2003)

From research performed during the first coronavirus outbreak in 2003, several peptide-based antagonists were also developed and tested and laid much of the groundwork for antagonists developed and studied during the current 2019–2020 pandemic (i.e., EK1). It seems, however, that none have made it into clinical trials or have been further advanced. Whether this occurred because of lack of funding, interest or unpublished negative results in additional studies in unknown. Below we summarize the main peptide-based antagonists targeting the ACE2–S protein interaction from the 2003 epidemic, which are listed in Table 1.

Heptad repeat regions (HR1 and HR2) of the S protein in SARS-CoV were identified as essential elements for viral entry. They help the SARS-CoV virus attach to the ACE2 receptor; this process leads to membrane fusion of the virus and the host cell. Xu *et al.* [39] studied the crystal structure of the SARS-CoV S protein fusion core and identified HR2-18 peptide, which comprises residues 1161–1187, derived from the HR2 region and binds to the relatively deep grooves on the surface of the central coiled coil. In another study, Yuan *et al.* [40] designed and identified 25 synthetic peptides; two of these peptides corresponded to the HR regions, HR1-1 and HR2-18, were identified as inhibitors, with EC_50_ values of 0.14 and 1.1 μM, respectively. The potency of these peptides was studied by wild-type SARS-CoV assay, and only one peptide, HR2-18, was found to inhibit the entry of the SARS-CoV with low activity. As expected, varying the amino acid sequences, addition or deletion of amino acid units found to be detrimental for the inhibition activity. On the other side, Han *et al.* [41] performed alanine-scanning mutagenesis analysis to identify then design peptides that resembled residues critical for entry mediated by the ACE2 receptor. They tested the activity of six synthetic peptides analogous to significant segments in ACE2. P6, which is a peptide comprised two discontinuous fragments, aa 22–44 and 351–357 connected by glycine (Table 1). This peptide potently inhibited SARS pseudovirus infection with an IC_50_ of approximately 0.1 μM. However, this peptide has not been studied in an animal model yet. Sainz *et al.* [42] studied and identified novel peptides just outside the HR sequences between the N- and the C-helical HR domains. Two peptides labeled SARSWW-III and SARSWW-IV were recognized within the S2 subunit corresponding to the aa residues 1028–1049 and 1075–1093, respectively. Based on the Wimley–White interfacial hydrophobicity scale (WWIHS), both peptides show potent inhibition for the viral infectivity with 5.7 and 7.1 points, respectively. The authors reason the inhibition effect to conformational changes required by the S2 protein during the fusion event. This group demonstrated that SARSWW-III and SARSWW-IV inhibited viral plaque formation on Vero E6 cells by 90 and 83%, respectively, at peptide concentrations of ∼30 μM. Such findings establish solid foundations for the development of new fusion peptide inhibitors related to the HR-adjacent regions. These studies spotlight the HR region as a critical target for the viral entry. These initial investigations have laid the groundwork for current research in targeting membrane fusion related sequences in the S2 region that could be potent viral entry inhibitors or significant functional probes.

In a quest for potent small molecules that inhibit the SARS-CoV entry to the human cells, Struck *et al.* [43] recognized the focal point of the viral protein that is necessary for the binding to the human cells. They identified RDB-11b (Table 1), a hexapeptide (438YKYRYL443) in the RBD of the S protein of SARS-CoV that binds to the human cell with recognizable affinity and specificity. RDB-11b found to reduce the viral infection of the epithelial cells in the upper respiratory tract by a factor of 600. In a comprehensive screening for viral entry inhibitor peptides of the SARS-CoV BJ01, Hu *et al.* [44] recognized S_471–503_, a peptide that overlaps the RBD of SARS-CoV. This peptide inhibits the viral entry by blocking the binding between the S protein RBD and the ACE2 receptor with high specificity. S_471–503_ could inhibit the SARS-CoV infection of Vero cells *in vitro* with an IC_50_ of 4.0 μM (with an EC_50_ value of 41.6 μM). After a few months, Ho *et al.* [45] reported another inhibitory peptide derived from S protein, named SP-10 (aa residues 668–679). Using a biotinylated enzyme-linked immunosorbent assay, SP-10 was found to significantly inhibit the binding of SARS-CoV S protein to ACE2, with an IC_50_ value of 1.88 ± 0.52 nM. Further analysis by immunofluorescence assays and S protein-pseudotyped retrovirus infectivity indicated that SP-10 can block both interactions of S protein and the infectivity of S protein-pseudotyped retrovirus with Vero E6 cells. These peptide-based inhibitors provided the theoretical basis for the work done on SARS-CoV-2 and have the potential to lead to potent cell fusion inhibitors against coronaviruses.

## Other potential peptide therapeutics for SARS-CoV-2

As previously mentioned, endogenous ACE2 peptide substrates are also possible candidates for peptide-based therapeutics, however, there has been some controversy with their potential use and side effects [49]. Recombinant human ACE-2 (RhACE2) as a therapeutic for active disease was being tested in a small Phase II clinical trial to evaluate its efficacy in SARS-Co-V-2 in China earlier this year, however the study was withdrawn for unspecified reasons (NCT04287686). This protein (RhACE2-APN01) is now being tested in Phase II at multiple sites in three countries (Germany, Denmark and Austria) with initial results expected by September 2020 (NCT04335136).

Proteases involved in both viral entry (host TMPRSS) and viral replication/transcription (3C-like protease M or M^pro^) have also been studied as a potential target for SARS-CoV-2 therapeutics [9,50]. One group has also found previously FDA-approved drugs that target viral M^pro^ inhibition that have shown some promise in preclinical and clinical studies [51], while others have worked on peptidomimetic candidates that target M^pro^ with some success [52]. All of these targets and therapeutics require further investigation and optimization but seem to also have significant potential in SARS-CoV-2.

## Discussion & conclusion

In the immediate future, repurposed drugs and vaccines will be the first line of defense against COVID-19 [53], however drug resistance and other issues with vaccines (use in immunocompromised or immune deficient individuals) should allow room for development of therapeutics targeting other pathways. Peptide-based therapeutics present several advantages over traditional small-molecule drugs, including increased specificity and both cost and time efficiency in synthesis. Although monoclonal antibodies also achieve this increased specificity, they require more costly and labor-intensive synthesis methods. In addition, there has been some controversy over possible antibody-mediated viral entry mechanisms, which could exacerbate disease [54]. The significance of the ACE2–S protein interaction in SARS-CoV-2 disease is currently under investigation using soluble RhACE2 (APN01) in Phase II clinical studies overseas. Other preclinical studies with S protein-targeted neutralizing antibodies and peptide-based inhibitors reviewed herein also provide strong evidence for the significance of this target in SARS-CoV-2. Going forward, preclinical efforts for screening of peptide-based inhibitors should be performed in *in vitro* models [43], before being tested in mice with humanized ACE2 [55], leading to further therapeutic development.

## Future perspective

The field of peptidomimetics has opened a new door for the field of peptide-based therapeutics. The potential for equivalent or even enhanced biological efficacy, extended bioavailability, and highly efficient synthesis and screening mechanisms, positions these drug candidates as attractive options for development in disease outbreak situations. The COVID-19 pandemic has benefitted from much of the research knowledge gained during the previous 2003 SARS-CoV outbreak. Similarities and differences among these two related viruses (SARS-CoV and SARS-CoV-2) in specific biological interactions at the peptide level can be used to develop peptidomimetic-based drug candidates to target the viral spike (S) protein-host receptor (ACE2) interaction.

**Executive summary**

**Potential of peptide-based therapeutics in disease & pandemic situations**

- Rapid, effective and cost efficient solutions are needed. Drug repurposing and immunotherapies (including passive and active immunization) are the first line of defense and lessons learned from these strategies assist the efficient design and development of peptide-based therapeutics.

**COVID-19/SARS-CoV-2**

- Lessons learned from related coronaviruses including SARS-CoV (2003) have been applied to the current pandemic. Detailed structural analyses of the surface viral spike (S) protein and the main host entry receptor, angiotensin-converting enzyme-2 (ACE2), have been used to develop potential peptide-based therapeutics. Further studies in animal models and clinical settings are now underway.

**Peptidomimetics**

- These synthetically modified peptides have great potential for advancement due to their enhanced stability and bioavailability. Several S protein- and ACE2-targeted peptidomimetics have been developed and are in various stages of development and validation.
